# A Novel Percutaneous Technique for Coaxial Treatment of Large Coronary Vessel Perforations—The RIP (Rip and Inflate in Perforations) Technique

**DOI:** 10.3390/jcm15083163

**Published:** 2026-04-21

**Authors:** Maximilian Will, Konstantin Schwarz, Gregor Leibundgut

**Affiliations:** 1 Department of Internal Medicine 3, University Hospital St. Pölten—NOE LGA, Karl Landsteiner University, Dunant-Platz 1, 3100 St. Pölten, Austria; konstantin.schwarz@stpoelten.lknoe.at; 2 Klinik für Kardiologie, Universitätsspital Basel, 4031 Basel, Switzerland; gregor.leibundgut@usb.ch

**Keywords:** percutaneous coronary intervention (PCI), coronary perforation, endovascular complications

## Abstract

**Background/Objectives**: Coronary perforations are infrequent but potentially fatal complications during percutaneous coronary intervention (PCI). Interventional management aims to stop extravasation and restore distal flow to prevent tamponade and cardiogenic shock. In current practice, the ping-pong technique is recommended to ensure sealing of the perforation during covered stent delivery. However, this method is complex, time-consuming, and requires a second vascular access. Therefore, we developed a technique that seals the perforation and enables covered stent implantation using a single guide catheter. **Methods**: This technical note describes a novel technique in which a guide extension catheter (GEC) can be advanced across a vascular perforation after balloon inflation. The insertion of the GEC is made possible by detachment of the balloon hypotube. To minimize leakage, a regular coronary wire introducer needle is attached to the snapped hypotube after GEC loading and continuously inflated to hold nominal pressure. Advancement of the GEC across the perforation immediately limits hemorrhage and facilitates covered stent deployment via a single vascular access. The technique was first evaluated in bench testing and subsequently applied in three illustrative clinical cases at a tertiary referral center using standard, commercially available devices. **Results**: Bench testing confirmed the reproducibility of the ripping maneuver and successful ballon inflation over enough time to advance the GEC with the introducer married with the ripped hypotube. In all clinical cases, the GEC was successfully advanced across the perforation, allowing prompt covered stent deployment where necessary using a single guide catheter and access site without technical failure. **Conclusions**: The RIP (Rip and Inflate in Perforations)—technique is a feasible and reproducible alternative to the ping-pong technique. Bench validation and initial clinical application suggest that it may simplify the management of large-vessel perforations while reducing procedural complexity and the need for additional vascular access.

## 1. Introduction

Coronary perforations during percutaneous coronary interventions remain a rare but life-threatening complication [1,2]. Their prevalence in routine PCI is reported to be approximately 0.2–0.5% [1] However, substantially higher rates are observed in complex procedures, particularly in chronic total occlusion (CTO) interventions (4%) [3], and these rates rise further with the use of retrograde techniques (up to 15%) [4]. Generally, coronary perforations can be categorized into three distinct types: large-vessel perforations, distal vessel perforations and collateral perforations [5]. Distal guidewire and septal collateral perforations are often amenable to balloon tamponade and fat or coil embolization, and typically pose a lower risk of immediate hemodynamic compromise than large-vessel perforations [6]. Epicardial collateral perforations may be more difficult to manage and often require more complex interventions with coiling from both sides [3,7]. Large-vessel perforations most commonly result from balloon or stent oversizing and from high-pressure inflation, particularly in tight, heavily calcified lesions that have not been adequately modified beforehand, including by rotational atherectomy [8]. Despite their low incidence, they carry substantial morbidity and mortality, particularly when involving complex and heavily calcified lesions [1,2]. Ongoing contrast extravasation may rapidly result in pericardial effusion, cardiac tamponade, and hemodynamic collapse. Therefore, immediate recognition and decisive management are essential. Contemporary interventional strategies aim to promptly achieve hemostasis and restore distal coronary perfusion to prevent tamponade, cardiogenic shock, and ischemia. Commonly, the ping-pong technique [9] is employed, requiring the use of two guiding catheters to reduce blood loss when delivering a covered stent. While effective, this method requires a second access site, can be complex and time-consuming, and may be challenging for non-CTO-PCI operators, as it is rarely performed during standard PCI procedures. Consequently, alternative bailout strategies that can be rapidly performed through a single access using standard PCI equipment are warranted. Here, we propose a technique that maintains sealing of the perforation while allowing covered stent implantation without the need for a second guiding catheter.

## 2. Materials and Methods

### 2.1. Study Design and Setting

This technical note was designed to describe a modified balloon-assisted technique for the advancement of a GEC across a vascular perforation and subsequent covered stent deployment. The technique was applied in three illustrative clinical cases treated at a tertiary referral center from January to November 2025 following extensive bench testing. The primary objective was to demonstrate technical feasibility and reproducibility of the approach rather than to evaluate clinical outcomes or comparative efficacy.

### 2.2. Materials and Equipment

All procedures were performed using standard, commercially available devices routinely used in interventional practice. The materials included a balloon catheter with a compatible hypotube, an introducer needle with an inner diameter matching the outer diameter of the balloon hypotube, a guide extension catheter, a standard guidewire, an indeflator device, and a covered stent graft. No custom-made or experimental equipment was used. Device selection and sizing were performed at the operator’s discretion according to anatomical and procedural requirements.

### 2.3. Technical Procedure

**Figure 1:**
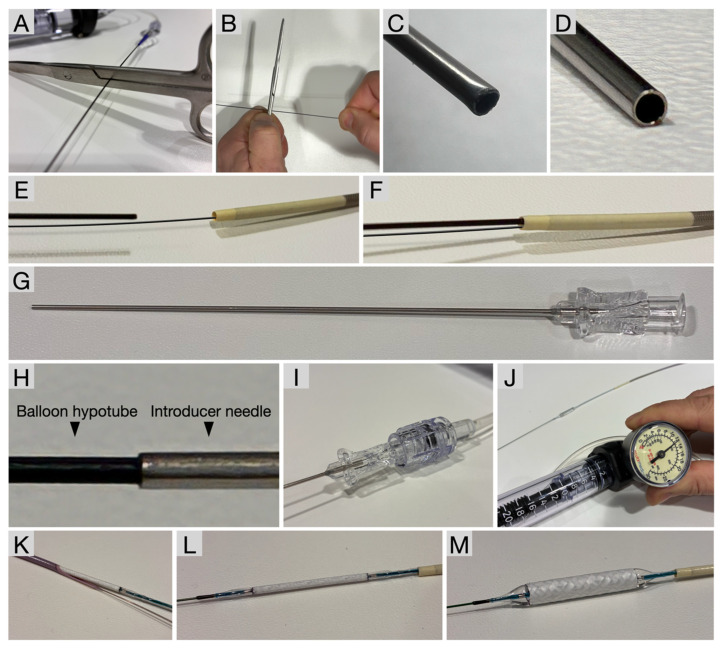
**RIP Technique—Step by Step.** (**A**). **Clamp the balloon hypotube** firmly using the backend of scissors. (**B**). **Rip the hub of the inflated balloon** by pulling and wiggling to achieve clean separation. (**C**). This **ripping creates a flush rupture** of the hypotube, preventing bending or deformation. (**D**). **Use an introducer needle whose inner diameter matches the hypotube’s outer diameter** for a tight fit. (**E**). **Advance the guide extension catheter (GEC)** over the ripped balloon hub and guidewire. (**F**). **Track the GEC toward the balloon.** (**G**). **Seat the introducer needle onto the ripped balloon hub,** pushing the hypotube into the needle as far as possible. (**H**). The **introducer needle will fit securely** on a properly ripped (non-bent) hypotube. (**I**). **Connect the introducer needle to the indeflator device.** (**J**). **Continuously inflate** the balloon, accepting contrast leakage at the needle–hypotube interface. (**K**). Once the **GEC is safely advanced beyond the perforation site** using an inchworming technique, **deflate and retract the balloon.** (**L**). **Advance the stent graft** to the site of rupture. (**M**). **Deploy the stent graft successfully**.

The described technique was developed to treat large vessel perforations by facilitating advancement of a GEC across a vascular perforation and subsequent deployment of a stent graft. Individual steps of this technique are illustrated in Figure 1.

First, the hypotube of an inflated balloon catheter was firmly clamped using the backend of surgical scissors. The balloon hub was then intentionally ruptured by controlled pulling and wiggling, resulting in a clean and flush separation of the hub from the hypotube. This maneuver was performed carefully to avoid bending or deformation of the hypotube, which is essential for subsequent steps.

An introducer needle was selected such that its inner diameter closely matched the outer diameter of the balloon hypotube to ensure a tight and stable connection. The guide extension catheter was advanced over the guidewire as well as the ripped balloon hub. Subsequently, the introducer needle was seated onto the ripped balloon hub, and the hypotube was advanced into the needle lumen as far as possible to achieve a secure fit. Proper alignment of the hypotube without bending was confirmed to ensure stability of the connection.

The introducer needle was then connected to an indeflator device, and the balloon was continuously inflated. Controlled contrast leakage at the needle–hypotube interface was accepted and did not interfere with balloon inflation or catheter advancement. Using an inchworming technique, the guide extension catheter was advanced beyond the perforation site while maintaining balloon support. After successful positioning of the guide extension catheter, the balloon was deflated and retracted.

Finally, the stent graft was advanced through the guide extension catheter to the site of rupture and deployed according to standard procedural practice.

### 2.4. Technical Success and Feasibility Assessment

Technical success was defined as the successful completion of the procedure using the proposed technique without technical failure. Feasibility was assessed qualitatively based on ease of integration into the existing workflow, and the need for additional technical adaptations. Procedural observations were documented descriptively without formal quantitative analysis.

### 2.5. Safety Considerations

Procedure-related complications and technical difficulties were monitored during and immediately after the procedure. Safety assessment was limited to descriptive reporting of adverse events related to the technical aspects of the procedure. No additional risks beyond those associated with standard PCI techniques were anticipated.

### 2.6. Ethical Considerations

The technical application described in this note was performed as part of routine clinical care. Ethical approval was obtained from the local institutional review board, and written informed consent was obtained from all patients prior to the procedure.

## 3. Results

Bench testing demonstrated stable coupling between the ripped balloon hypotube and the introducer needle, allowing continuous balloon inflation despite controlled contrast leakage at the needle–hypotube interface. Advancement of the guide extension catheter over the modified balloon system was reproducible under simulated conditions.

Following bench validation, the technique was applied in three clinical bailout cases involving acute, life-threatening vascular perforation. In all cases, the guide extension catheter was successfully advanced beyond the perforation site using the described technique, enabling subsequent delivery and deployment of a covered stent graft if it was necessary. No technique-related device failures were observed. Minor case-specific adjustments were required to accommodate anatomical and procedural differences but did not affect technical feasibility or procedural completion.

### 3.1. Clinical Cases

#### 3.1.1. Case 1—Coronary Perforation of the Proximal Left Anterior Descending Artery (LAD)

**Figure 2:**
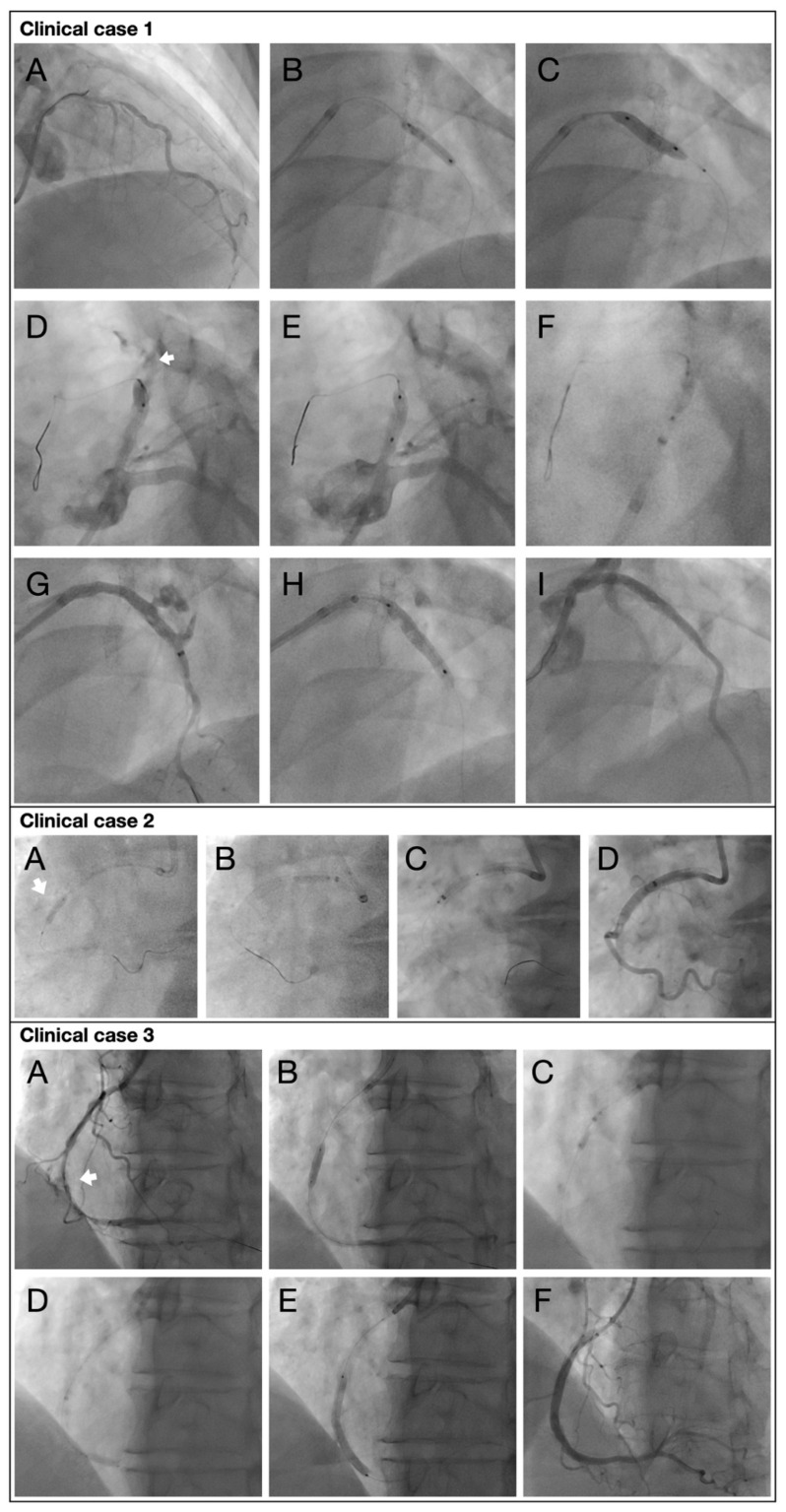
**Clinical case scenarios. Clinical case 1.** Perforation of the proximal left anterior descending artery (LAD). (**A**). Severely calcified proximal LAD lesion prior to intervention. (**B**). Initial balloon dilatation with insufficient lesion preparation. (**C**). Escalation of calcium modification using a cutting balloon to enable adequate lesion preparation. (**D**). Angiographic evidence of coronary perforation following stent implantation. (**E**). Immediate inflation of the stent-balloon proximal to the perforation site to achieve temporary hemostasis. (**F**). RIP technique enabling advancement of the GEC while maintaining balloon inflation. (**G**). Advancement of the GEC over the perforation site. (**H**). Deployment of a covered stent to definitively seal the perforation. (**I**). Final angiographic result after additional high radial-force stent implantation, demonstrating complete sealing of the perforation and optimal stent expansion. **Clinical case 2**. Perforation of the proximal RCA. (**A**). Blocking the perforation site with the RIP balloon. (**B**). “Inchworming” the GEC at ostium due to sharp bend and fresh stent. (**C**). Advancement of GEC by several inchworm maneuvers. (**D**). Final contrast injection through the GEC was performed after effective temporary sealing with the “RIP balloon” for approximately 2 min. **Clinical case 3**. Perforation of the mid RCA. (**A**) Angiographic evidence of coronary perforation. (**B**) Placement of a blocking balloon using the RIP technique to achieve temporary sealing of the perforation. (**C**) Advancement of the GEC by repeated “inchworming” maneuvers facilitated by the RIP balloon. (**D**). Distal anchoring of the guide extension catheter using the RIP balloon to provide additional support. (**E**). Stent deployment for definitive treatment of the perforation under maintained vessel sealing. (**F**). Final angiographic result demonstrating successful perforation closure and restoration of coronary flow.

A 56-year-old patient with chronic coronary syndrome underwent PCI of the proximal LAD for a severely calcified lesion (Figure 2, Case 1, A). After initial balloon dilatation and insufficient lesion preparation, calcium modification was escalated using a cutting balloon (Figure 2, Case 1, B and C). This allowed adequate lesion modification and implantation of a drug-eluting stent (DES). Angiography subsequently revealed a coronary perforation in the proximal LAD. The stent-balloon was immediately positioned slightly proximal to the perforation site and inflated at nominal pressure to achieve temporary hemostasis (Figure 2, Case 1, D and E). Contralateral left radial access for a ping-pong technique was not feasible due to severe radial spasm, and femoral access was unfavorable due to advanced peripheral artery disease. Subsequently, the proximal segment of the hypotube of the balloon catheter was clamped between the shanks of a pair of scissors. The hypotube was then gently moved up and down while pulling, generating a flush rip of the hypotube without deformation of the outer diameter, allowing the hypotube to be inserted into the introducer needle after advancement of the guide extension catheter (GEC) over the guidewire and the ripped hypotube (Figure 2, Case 1, F and G). Immediately after connecting the introducer needle to the indeflator, the balloon was reinflated to keep occluding the perforation site. Once the GEC reached the balloon, the balloon was deflated to allow advancement of the GEC over the balloon. This maneuver partially sealed the perforation and facilitated the delivery of a covered stent to the perforation site. (Figure 2, Case 1, H). Despite immediate measures, the patient developed pericardial tamponade requiring urgent ultrasound-guided subxiphoid pericardial drainage. A total of 500 mL of blood was evacuated, resulting in immediate hemodynamic stabilization. Despite repeated postdilatation and intravenous administration of protamine to reverse heparin anticoagulation, a proximal endoleak persisted but was eventually sealed after further balloon optimization. Subsequently, a complete collapse of the stent in the proximal LAD was observed. Multiple postdilatations resulted in acute recoil. Definitive stabilization was achieved only after implantation of an additional high radial-force stent, resulting in optimal stent expansion and angiographic outcome (Figure 2, Case 1, I). The patient was transferred to the general ward in stable and asymptomatic condition. The pericardial drain was removed on the following day, and the patient’s subsequent clinical course remained uneventful.

#### 3.1.2. Case 2—Perforation of the Proximal Right Coronary Artery (RCA)

In a second case, a 72-year-old female patient with CCS developed a perforation of the mid RCA during CTO-PCI. The previously described bailout technique was immediately applied, aiming for continuous control of the perforation and rapid preparation for definitive sealing. Hence, the perforation site was promptly occluded with the used balloon, effectively limiting extravasation while maintaining procedural control (Figure 2, Case 2, A). Under these stabilized conditions, a GEC was advanced to cover the inflow and perforation site (Figure 2, Case 2, B) and to potentially enable delivery of a covered stent (Figure 2, Case 2, C and D). Due to a sharp ostial bend and the presence of a freshly implanted stent, advancement of the GEC required repeated “inchworming” maneuvers, initially at the ostium and subsequently in a stepwise fashion toward the target segment. Although GEC can facilitate easier stent-graft delivery in difficult anatomies, in the present case covered stenting was not required, as the “RIP balloon” provided an effective definitive seal after occluding the vessel for approximately two minutes. The subsequent clinical course was uneventful, and the patient was discharged in stable condition.

#### 3.1.3. Case 3—Perforation of the Mid Right Coronary Artery

An elective PCI of the RCA was performed in a patient with known severe stenoses extending from the ostial to the distal vessel. After predilation using a 4.0 mm non-compliant balloon, coronary perforation occurred in the mid-segment, accompanied by acute hypotension, chest pain, and loss of distal flow (Figure 2, Case 3, A). Immediate balloon tamponade of the affected segment was performed (Figure 2, Case 3, B). Again, the RIP technique enabled advancement of a GEC using inchworming maneuvers facilitated by the “RIP-balloon” (Figure 2, Case 3, C). After distal anchoring with the “RIP-balloon” it was possible to deploy two drug-eluting stents from the distal to the ostial RCA (Figure 2, Case 3, D and E). Repeated echocardiographic assessments excluded the presence of a pericardial effusion. Final post-dilatation was carried out with a 4.0 mm non-compliant balloon at pressures up to 20 atm, resulting in an optimal angiographic and intravascular imaging result (Figure 2, Case 3, F). The patient experienced an uneventful clinical course and was discharged in stable condition.

## 4. Discussion

This novel percutaneous technique for coaxial treatment of large vessel perforations offers an efficient and less invasive approach to treating coronary perforations during PCI using a single access. Due to the unique ripping technique with the hypotube and the ability to quickly reinflate the balloon in the perforation we call it the RIP (Rip and Inflate in Perforations) technique.

This approach may be particularly valuable when only a single vascular access site is available, a situation more likely in elderly patients and those with comorbidities such as peripheral artery disease and renal insufficiency, conditions commonly associated with extensive vascular calcification. In these patients, the risk of coronary perforation is typically elevated [8,10].

The traditional ping-pong technique [9], while effective, is not frequently performed during standard PCI procedures, leading to operator reluctance. This method requires the insertion of a second guiding catheter via an additional vascular access site. The first guiding catheter must be slightly retracted out of the ostium to enable the engagement of the second catheter while the blocking balloon remains inflated. Through the second catheter, a second coronary guidewire is advanced to the occlusion site, followed by a brief deflation of the blocking balloon to allow the guidewire to pass into the distal vessel segment. Once positioned, a covered stent is advanced over this second guidewire. As the covered stent reaches the balloon position, the balloon is carefully deflated and withdrawn to allow precise stent graft deployment.

While this technique can be effective, many operators may prefer to deflate the balloon and deliver the covered stent directly through the initial guiding catheter due to its simplicity. However, minimizing the balloon deflation time is critical to reduce the risk of blood extravasation into the pericardium and the subsequent development of cardiac tamponade [8]. In some cases, the delivery of the covered stent can be achieved through the same guiding catheter alongside the blocking balloon with minimal deflation time, referred to as the “block and deliver technique” [11]. In comparison, the “block and deliver” technique typically requires the use of 8F guiding catheters to allow simultaneous passage of a blocking balloon and a covered stent, which are not routinely used in contemporary practice and typically necessitate femoral access. Although this approach is technically straightforward and does not require device modification, it involves parallel manipulation of two devices over two guidewires, and interaction between the balloon and the covered stent within the vessel segment and the guiding catheter may be challenging. These limitations are similar to those encountered with the ping-pong technique.

By employing the RIP technique, a similar approach is possible using smaller guides. The necessity of a second access site and a guiding catheter with the ping-pong technique is entirely avoided. Deploying a guide extension over the rupture site offers multiple benefits. While it may decrease the degree of extravasation, it also facilitates the implantation of a covered stent graft without the risk of interference from calcification, angulations, or previously placed stents, particularly in cases of vessel rupture during postdilation.

An additional advantage of the RIP technique is the ability to repeatedly inflate and deflate the balloon, enabling controlled inchworming of the GEC in challenging anatomies. Use of the GEC further mitigates the risk of hydraulic dissection at an unstented ostium.

The RIP technique will most commonly be applied in 6F or 7F guiding catheter systems. In the context of 7F access, the selection of a 6F or 7F guide-extension catheter should be individualized based on vessel size, coronary anatomy, and the location of the perforation.

Although the nominal inner diameter of a standard 0.014 PCI introducer needle (≤0.46 mm) and the outer diameter of the proximal balloon catheter shaft (≈0.6 mm) are closely matched, practical compatibility is achieved through manufacturing tolerances, the chamfered entry of the shaping needle, and the compliant nature of the balloon catheter shaft. Depending on the balloon configuration and the exact location of the hypotube detachment, the system may not be completely leak-proof. Consequently, continuous manual compensation by a second operator is required to maintain stable balloon pressure. In this case, as well as in multiple prior bench evaluations, the pressure could be maintained above nominal levels, and contrast loss over a 1 min inflation period consistently remained below 10 mL.

Rapid advancement of the GEC while continuously occluding the perforation site provides maximal control and secure access to the perforation. In selected cases, prolonged balloon inflation alone may be sufficient to achieve definitive sealing.

A dedicated device for the management of coronary perforations is the Ringer™ perfusion balloon catheter (Teleflex) [12]. It allows for prolonged inflation up to one hour by maintaining distal perfusion through a central lumen while simultaneously controlling bleeding at the perforation site. However, the placement of the Ringer balloon requires the removal of the initial balloon that caused the rupture. Furthermore, prior to covered stent delivery, the Ringer balloon itself must also be withdrawn, thereby interrupting occlusion again. In contrast, the RIP technique addresses the need for stent delivery during occlusion. It effectively minimizes blood loss, streamlines the procedure, and enables a smooth transition from temporary hemostasis to definitive stenting.

### Limitations

Several limitations should be acknowledged. Importantly, successful execution of this technique requires precise hypotube detachment, as irregular edges may compromise compatibility. This aspect can be addressed through adequate prior bench testing and familiarization with the technique. While this maneuver has been successfully performed in bench testing and in isolated clinical cases, it has not yet been systematically validated across larger case numbers, all balloon sizes, or the full range of commercially available guidewire shaping introducers. Device dimensions and design characteristics may vary between manufacturers, further limiting generalizability. These factors underscore the importance of prior bench testing and local familiarization with the specific devices in use before clinical application in emergency situations.

In cases of challenging GEC delivery, multiple repositioning attempts of the intentionally ruptured balloon may be required. This can necessitate repeated inflations with a partially leaking system, potentially requiring indeflator refilling and resulting in suboptimal balloon rewrapping, which may limit procedural efficiency.

## 5. Conclusions

The RIP technique represents a promising alternative to the traditional techniques, providing a sophisticated single-guide approach to managing coronary perforations during PCI. Further studies are warranted to confirm the safety and efficacy of this approach.

## Data Availability

The data presented in this study are not publicly available due to privacy and ethical restrictions but are available from the corresponding author upon reasonable request.

## Abbreviations

The following abbreviations are used in this manuscript:

CCS	Chronic coronary syndrome
CTO	Chronic total occlusion
GEC	Guide extension catheter
LAD	Left anterior descending artery
PCI	Percutaneous coronary intervention
RCA	Right coronary artery
RIP	Rip and inflate in perforations
